# Microwave-Assisted Water Extraction of Aspen (*Populus tremula*) and Pine (*Pinus sylvestris* L.) Barks as a Tool for Their Valorization

**DOI:** 10.3390/plants11121544

**Published:** 2022-06-09

**Authors:** Matiss Pals, Liga Lauberte, Jevgenija Ponomarenko, Maris Lauberts, Alexander Arshanitsa

**Affiliations:** Latvian State Institute of Wood Chemistry, Dzerbenes Street 27, LV-1006 Riga, Latvia; matiss.pals@kki.lv (M.P.); liga.lauberte@kki.lv (L.L.); jevgenijaponomarenko@inbox.lv (J.P.); arshanica@edi.lv (A.A.)

**Keywords:** bark, microwave-assisted extraction, salicin derivatives, oligomeric proanthocyanidins

## Abstract

The barks of aspen (*Populus tremula*) and pine (*Pinus sylvestris)* are byproducts of wood processing, characterized by their low economic value. In the present study, microwave-assisted one-cycle water extraction was explored as a tool for the valorization of this biomass as a source of biologically active compounds. The microwave extractor of the original construction equipped with a pressurized extraction chamber and a condenser section was used. The microwave-assisted extraction (MAE), specially including dynamic dielectric heating up to 70 °C followed by 30 min of isothermal heating, promoted the isolation of salicin from aspen bark, allowing for the obtention of a two-times-higher free salicin concentration in water extracts (−14% vs. 7%) reached by multi-cycle accelerated solvent extraction (ASE), which is an advanced technique used as a reference. The MAE of pine bark with dynamic heating up to 90–130 °C, avoiding the isothermal heating step, allowed for the obtention of a 1.7-times-higher concentration of proantocyanidin dimers-tetramers, a 1.3-times-higher concentration of catechin and a 1.2-times-higher concentration of quinic acid in water extracts in comparison to a more time- and solvent-consuming ASE performed at the same temperature. The biological activity of the obtained extracts was characterized in terms of their ability to inhibit xahntine oxidase enzyme, which is a validated target for the therapeutic treatment of hyperuricemia.

## 1. Introduction

Plant biomass research nowadays is focused on the conversion of its processing residues into products with a minimum amount of waste by using the biorefinery concept. The bark comprises about 5–20% of the wood, and its annual production as a byproduct of the chemical or mechanical processing of forest biomass is estimated to be around 300–400 million m^3^ globally [1,2]. In the vast majority of cases, it is simply left in the forest or burnt, recycled as a fuel source or insulation material or used in horticulture for weed suppression and moisture retention. Yet, it is important for sustainable development that each type of biomass, including bark, is used in a cascading manner, primarily as a raw material for the production of high value-added substances, with the utilization of the remaining biomass wastes as a source of solid, liquid or gaseous fuels available for heat and electricity generation [3]. Extraction is regarded as a key technology for valorizing bark as a source of multipurpose products. It has a small output but is characterized by a high added value [4,5,6]. Due to the accumulation of biologically active compounds in the bark of trees, which results from plant evolution as a response to biotic and abiotic stresses, the bark-derived extracts have interesting pharmacological and cosmetic activities [1,7]. The composition and properties of bark extracts depend on tree species as well as on the extraction technique and the extraction solvents that are used [8]. Extraction methods relying on large volumes of organic solvents are not easily scaled to the quantities needed for the large-scale processing of timber by-products. Moreover, solvent evaporation requires ventilation devices and specialized respiratory equipment in industrial setting studies [9,10]. Further, the use of organic solvents for natural products isolation contradicts the major principles of “green chemistry”, which require the invention and design of processes to eliminate the use and generation of hazardous substances [11]. The use of alternative, environmentally friendly solvents such as water is the basic specification for the development of novel extraction processing.

Among the compounds that can be extracted from tree bark by water are salicin (salicyl alcohol glucoside) and other phenolic glucosides, as well as salicylic acid and its derivatives occurring in the bark of trees of the family *Salicaceae* as willow, poplar and aspen. They have antipyretic, analgesic, anti-inflammatory, antirheumatic and anticoagulant effects in humans and animals. Due to the presence of these compounds, willow bark has been used in medicine for millennia to alleviate pain and fever [3]. The willow species *Salix purpurea*, *Salix daphnoides*, *Salix fragilis*, *Salix alba* and *Salix nigra* are used to manufacture pharmaceutical products in cosmetic applications. Salicin exhibits anti-irritative properties in topical dermatological applications. Salicin also shows an anti-aging effect on the skin. The addition of salicin to collagen film improves the material adhesion to the skin, which can be a valuable feature in biomaterial applications, along with the anti-inflammatory properties of salicin [12]. The European aspen (*Populus tremula*) is widely used in the production of cellulose, chips and plywood. In most of these processes, debarking takes place, which, together with targeted products, produces a considerable amount of waste in terms of discarded tree bark [13,14]. Aspen bark can be considered as a potential source of salicin and salicylates. For example, due to the high content of salicylates, American aspen (*Populus tremuloides*) bark water soluble extracts are commercialized as cosmetic components with skin conditioning and antimicrobial properties [15]. The application of the microwave-assisted extraction technique for the isolation of salicin and related compounds from the bark of European aspen (*Populus tremula*) can enhance the concentration of biologically active compounds in water extracts and promote its use as a source of valuable products for cosmetics and pharmaceutics. Microwave-assisted extraction is advantageous over conventional extraction techniques, with an improved efficiency that is based on the disruption of the plant cell walls, thus promoting the extraction of the bonded secondary metabolites, as well as on the direct impact on polar compounds [16].

Another highly valuable compound that can be extracted by hot water from tree bark is tannins. The traditional application for tannins is the treating of animal hide to make leather, thereby making it rot-proof, softer and more durable. There are also emerging applications of tannins as a replacement of phenol-based chemicals of fossil resource origination that are used traditionally for the production of wood adhesives that are widespread in the wood composites industry and for rigid foams for the automotive and building sectors [17]. Condensed tannins–proanthocyanidins can be divided into procyanidins and prodelphynidins. Procyanidins are composed of oligomers and polymers, consisting of (+)-catechin and/or (-)-epicatechin units linked mainly through C4 → C8 and/or C4 → C6 bonds (B-type). These flavan-3-ol units can be doubly linked by a C4 → C8 bond and an additional ether bond from O7 → C2 (A-type) [18,19]. Oligomeric procyanidins have been reported to have protective or preventative functions in eye disease, aging, cancer and cardiovascular and cerebrovascular diseases, and they are powerful antioxidants [20]. Pine is one of the plants with the highest content of procyanidins. Pycnogenol^®^, Flavangenol^®^ and Oligopin^®^ are trademarks of the commercially available extracts of maritime pine (*Pinus pinaster*) bark, which contains procyanidin dimers, trimers, oligomers and polymers, phenolic acids (e.g., protocatechuic, gallic, ferulic and caffeic acids), flavanones (e.g., taxifolin and taxifolin-3′-β-D-glucoside), etc. [19,21]. *Pinus sylvestris* L. bark is available as waste in large quantities in many countries and can be considered as an alternative source of high value-added products to *Pinus pinaster* bark [11,22]. Several constituents of pine bark extracts, such as gallic, protocatechuic acids and catechin, are readily adsorbed by human skin and make the preparations useful for topical application [19]. Synergisms between quinic acid and the phenolic compounds may have promoted the bioactivities of the pine bark polar extracts [21]. However, the high polymerization degree and high molecular weight of a significant part of bark proanthocyanidins make them unable to penetrate biological membranes effectively, limiting both their biological activity and range of applications [20]. Procyanidins in *Pinus sylvestris* L. were found to be from dimers to decamers and with a higher polymerization degree classified as procyanidin polymers [23]. Green solvents as water and water/ethanol mixtures are less effective than acetone/water/acetic acid mixtures (70:29.5:0.5, *v*/*v*/*v*) in extracting proanthocyanidin polymers from dried red grape pomace by accelerated solvent extraction at different temperatures. However they are exceedingly more effective in extracting procyanidin monomers, dimers, trimers, tetramers and pentamers [24]. The depolymerization of high-molecular-weight proanthocyanidins is considered as an approach allowing one to overcome the limitations connected with their extraction and application [16,25]. Microwave-assisted extraction, specially using 95% ethanol as a solvent, a temperature of 170 °C and 55 min of extraction duration, was shown to be more effective than the conventional extraction method for recovering monomeric catechins and proanthocyanidins from grape seed powder. Therefore, the proanthocyanidins-based product obtained from grape seed by MAE showed a higher antioxidant capacity and α-glucosidase-inhibitory activities than that of the conventional method [25].

The aim of this work was the intensification of the water extraction of biologically active compounds, with a focus on salicin and proanthocyanidins from the barks of Latvian trees as logging residues for the development of therapeutic agents for the cosmetics and health-care industries. To access the phenolic compounds bounded within plant cellular walls and promote the depolymerization of high-molecular proanthocyanins during bark water extraction, an original microwave-assisted extractor [26,27,28] with the regime of ‘’steam explosion’’ was used.

The objectives of this study:

- The evaluation of the effectivity of the one-step water MAE of aspen (*Populus tremula*) and pine (*Pinus sylvestris* L.) barks in terms of yields of extractives;
- The characterization of the composition of bark water extracts obtained by microwave-assisted water extraction and reference-accelerated solvent extraction;
- The characterization of the advantages and effectiveness of aspen and pine barks MAE in terms of the isolation of salicin derivatives, flavonoids, proanthocyanidins and quinic acid, depending on the extraction conditions;
- The characterization of the biological activity of the obtained extracts in terms of their ability to inhibit xanthine oxidase enzyme, which is a validated target for the therapeutic treatment of hyperuricemia caused by uric acid overproduction and which has also been associated with a variety of conditions such as diabetes, hypertension and other cardiovascular diseases [29].

## 2. Results and Discussion

### 2.1. Composition of Aspen (Populus tremula) and Pine (Pinus sylvestris *L.*) Barks and the Efficiency of Their Water Extraction

Bark refers to lignocellulosic biomass, which is mainly composed of carbohydrate polymers: cellulose, hemicellulose and aromatic polymer lignin. The total summary content of carbohydrates (represented mainly by cellulose and hemicellulose) in aspen and pine bark was evaluated by the content of monomeric sugars after hydrolysis. The content of lignin was evaluated according to the Klason procedure [30]. The minimum theoretical content of extractives was calculated assuming that the other constituents of bark, except for cellulose, hemicellulose and lignin, are ash and extractives (Table 1). In view of the raw material chemical utilization, the results showed that the major chemical components of the bark under study were notably lower in terms of carbohydrates content and slightly higher in terms of lignin content in comparison to hardwood and softwood, as, in general, the wood carbohydrates content is estimated to be >60% and the lignin content is estimated to be 19–28% [31,32].

The bark’s heating value as a fuel is determined by the content of carbon (C), hydrogen (H), oxygen (O) and nitrogen (N) [33]. Compared to oil products, the bark under study, as well as the biomass generally, has too little hydrogen, too much oxygen, a lower fraction of carbon and a significant amount of ash [6] (Table 2).

These data clearly indicate that aspen and pine bark cannot compete with fossil resources as an energy carrier, and, therefore, the finding of alternative pathways of more effective valorization is quite an urgent task. At the same time, bark differs chemically from wood, with an overall higher proportion of extractives, which can be considered as low-volume but high-value products [32,34,35,36]. The theoretical available content of extractives for aspen and pine bark was evaluated as at least 26.5 and 14.1%, respectively, representing the significant part of the bark biomass (Table 1). This allows for the proposal that the isolation of extractives for their non-energy utilization as high-value products, with the further utilization of the residual biomass for heat and energy production, will be more economically feasible than the direct combustion of raw bark.

**Figure 1:**
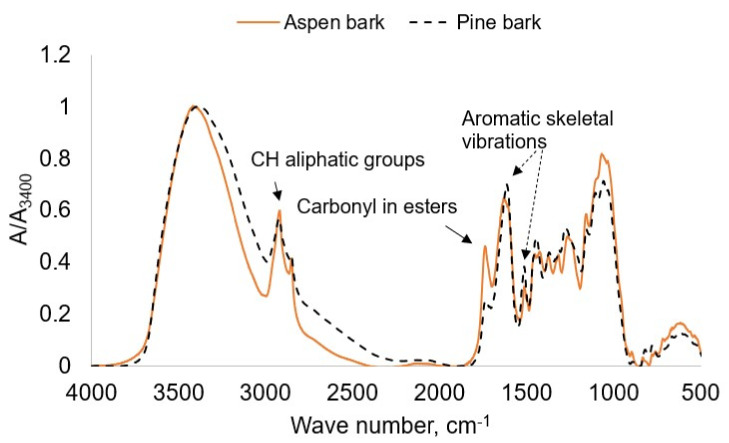
FTIR spectra of aspen and pine bark.

The chemical compositions of bark extractives differ depending on the tree species (Figure 1). It was shown that, in contrast to pine bark, aspen bark contains significant amounts of lipophilic compounds of suberin origination, which were revealed on FTIR spectra as vibrations of ester bonds and aliphatic CH groups (Figure 1).

**Figure 2:**
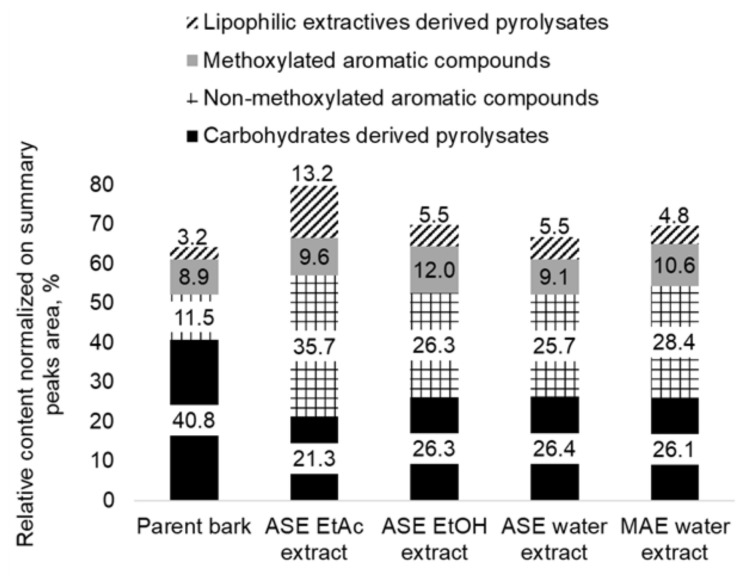
The composition of volatile pyrolysis products of parent aspen bark and its extracts (20 min of extraction at 70 °C) by different solvents and extraction techniques (the relative standard deviation for three repeated tests was ≤10%).

This work focuses on the valorization of bark as a source of biologically active aromatic hydrophilic extractives. The choice of water as a solvent and of microwave-assisted extraction with a higher selectivity to polar molecules as a technique allows for the reduction of the contamination of these products with suberin and other lipophilic compounds. This was confirmed by the data of the analytical pyrolysis of parent aspen bark and its extracts, which were obtained by different methods and solvents (Figure 2). The composition of bark and its extracts was characterized by categorizing volatile degradation products formed from biomass components during their thermal decomposition in conditions of analytical pyrolysis. The volatile pyrolysis products were classified as carbohydrate-derived compounds represented mainly by aliphatic acids and esters, aliphatic alcohols, aliphatic aldehydes and ketones, furan and pyran derivatives, cyclopentane derivatives, anhydro sugars, non-methoxylated aromatic compounds derived mainly from phenolic extractives, methoxylated phenols, guaiacyl (G-) and syringyl (S-) derivatives derived from lignin and lipophilic extractives derived from pyrolysates represented mainly by compounds with a long aliphatic chain such as dodecane, 1-nonene, octanoic acid, tetradecane, (Z)-3-tetradecene, pentadecane, 1-pentadecene, eicosane, (E)-9-eicosene, etc.

Water extraction allows for the concentration of hydrophilic aromatic compounds, which were revealed to be significantly increased in the relative content of non-methoxylated aromatic pyrolysis products in obtained extracts in comparison to parent bark. The efficiency of microwave-assisted water extraction (MAE) in terms of the isolation of aromatic compounds is close to that of organic solvents. Carbohydrates are the main compounds reducing the concentration of biologically active secondary metabolites in the water extracts (Figure 2).

**Figure 3:**
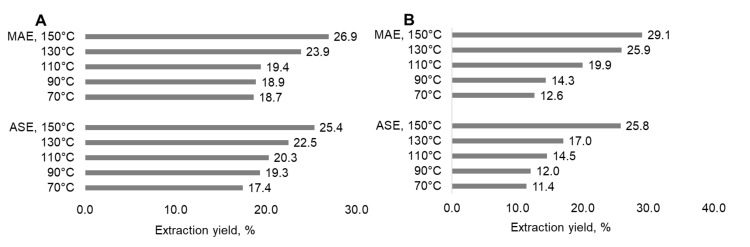
Water extraction yields on the dry matter (DM) of aspen (**A**) and pine (**B**) bark using the MAE and ASE methods (the relative standard deviation for three repeated experiments was ≤5.0%).

The yields of water-soluble European aspen (*Populus tremula*) bark extractives (on DM of biomass) isolated at 70–150 °C were close for both the MAE and ASE methods and varied in the ranges of 16.9–26.9%, steadily increasing with the increasing extraction temperature (Figure 3). The reported yields of the hydrophilic extractives isolated from the bark of aspen trees varied in a similar range of 15–24%, being close for different solvents such as acetone, water, methanol and ethanol (Table 3). The yields of the extractives isolated from the bark of pine (*Pinus sylvestris* L.) by water at 70–110 °C varied in the ranges of 11.4–14.5% for ASE and of 12.6–19.9% for MAE (Figure 3) in comparison to the range of 17.4–19.4% reported for the *Pinus sylvestris* bark extracted by acetone, water and alcohols (Table 3). The yields of pine bark extractives obtained at 130–150 °C by water MAE and at 150 °C by water ASE are higher: 25.8–29.1%.

Based on the obtained results, deionized water can be characterized as the most suitable solvent for the microwave-assisted extraction of tree bark, as it has a high microwave absorbance capacity, a high dissolution capability of barks’ phenolic metabolites and the absence of an environmentally harmful impact [32].

### 2.2. Composition of Aspen Bark Water Extracts

**Figure 4:**
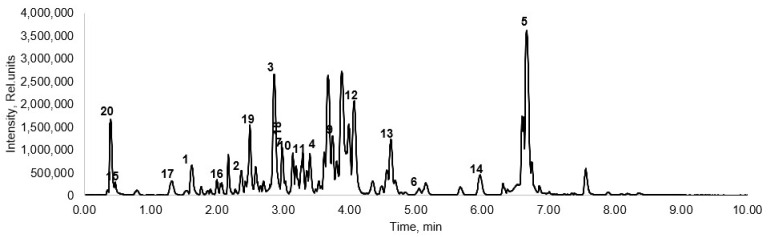
Total ion chromatogram of the aspen bark water extract obtained by microwave-assisted extraction at 90 °C, 30 min of isothermal heating (identification of compounds is presented in Table 4).

The qualitative composition of aspen bark water extracts was characterized by UHPLC-ESI-MS/MS (Figure 4). The results indicated that the major constituents of the trembling aspen bark water extracts are phenolic glycosides such as salicin and its derivatives, tremulacin, tremuloidin, kaempferol-hexoside, luteolin-hexoside, etc. (Table 4). Phenolic glycosides are phytochemicals known to be present at up to 30% of dry plant mass in the *Salicaceae* family. These secondary compounds function as defense chemicals against herbivores, including both insects and mammals [40,41]. Other phenolic constituents of aspen bark water extracts are phenolic acids and flavonoids (Table 4).

### 2.3. Effectivity of Water MAE in Terms of the Isolation of Salicin, Flavonoids and Proanthocyanidins from Aspen Bark

Salicin is a prodrug which is gradually hydrolyzed to aligenin by intestinal bacteria and converted into salicylic acid after absorption. Thus, it produces an antipyretic action without causing gastric injury [41]. The content of free salicin in aspen (*Populus tremula*, *Salicaceae* family) bark water extracts obtained by one-cycle MAE at different temperatures and different durations of the isothermal heating step varied in the range of 8.23–14.05% with a yield of 1.41–2.99% on bark dry matter (Table 5). The crude extract of salicin from the bark of *Salix alba* (*Salicaceae* family), which is used to manufacture pharmaceutical products (Chashai Kinglong Bioproducts, Changshai, China), contained 13.5% of salicin [41]. The maximum free salicin content in the aspen bark water extracts reached by accelerated solvent water extraction (ASE) was 7.4% with a yield 1.9% on the DM of bark by the application of the following extraction conditions: 150 °C, four extraction cycles of 5 min each (after each cycle of extraction, the extract was removed and a new portion of solvent was added, collecting the extracts together). The MAE promotes the isolation of free salicin from aspen bark, allowing for the obtention of a free salicin concentration in water extracts that is two times higher in comparison to that obtained by ASE. The content of salicylates in the tree bark, calculated as the total salicin determined after hydrolysis and reported for the Salix species, varies in the range of 0.14–12.06% depending on the tree species, the time of collection and the age of the plant [3,42]. According to the Monograph of the European Pharmacopoeia and the Evaluation Report of the European Medicines Agency, the potential plant feedstock, i.e., the bark of willow, should contain not less than 1.5% of salicin. The content of total salicin in the aspen bark water extracts obtained by MAE at different conditions varied in the range of 12.07–19.33% with a yield of 2.11–3.92% on the DM of bark (Table 5). This is higher in comparison to the *Salix alba clone*, *Salix daphnoides clone*, *Salix purpurea* and *Salix herbacea* methanol extracts obtained at 60 °C for three cycles of extraction, containing 1.7–9.6% of total salicin [43]. The maximum total salicin content in the aspen bark water extracts reached by ASE water extraction was 16.9% with a yield of 3.1% on bark dry matter by the application of the following extraction conditions: 70 °C, four extraction cycles of 5 min each. It was established that the maximum content of total salicin in both the MAE and ASE extracts is reached at 70 °C. However, the maximum free salicin content is reached at MAE extracts obtained at 110 °C vs. ASE extracts obtained at 150 °C. Thus, European aspen bark can be considered as a qualitative source of salicin derivatives, and its value can be enhanced by the application of MAE.

**Figure 5:**
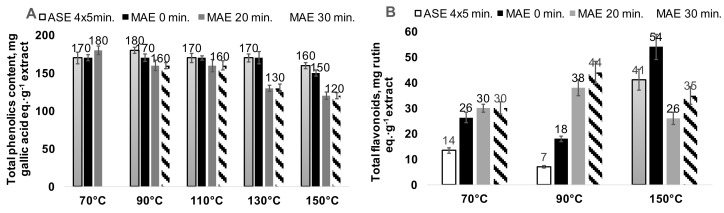
Total phenolics (**A**) and flavonoids (**B**) content in the aspen bark water extracts obtained at different conditions (the error bars present the standard deviation of three repeated tests).

The aspen bark water extracts obtained by one-cycle MAE and four-cycle ASE at 70–110 °C have close contents of total phenolics, varying in the range of 160–180 mg of gallic acid equivalents per g of extract. The increase in the MAE extraction temperature to 130–150 °C within 20–30 min of isothermal heating significantly reduced the total phenolics content in the water extracts (Figure 5A). The single cycle MAE extraction allows for the obtention of significantly higher contents of flavonoids in the aspen bark water extracts in comparison to the ASE performed at corresponding temperatures, despite ASE including the renewal of solvent (four cycles of extraction). The maximum total flavonoids content in the aspen bark water extracts reached by MAE—with high rate (25 °C·min^−1^) dynamic heating to 150 °C, avoiding the isothermal step—was 1.3 times higher in comparison to the maximum flavonoids content reached by ASE (Figure 5B).

**Figure 6:**
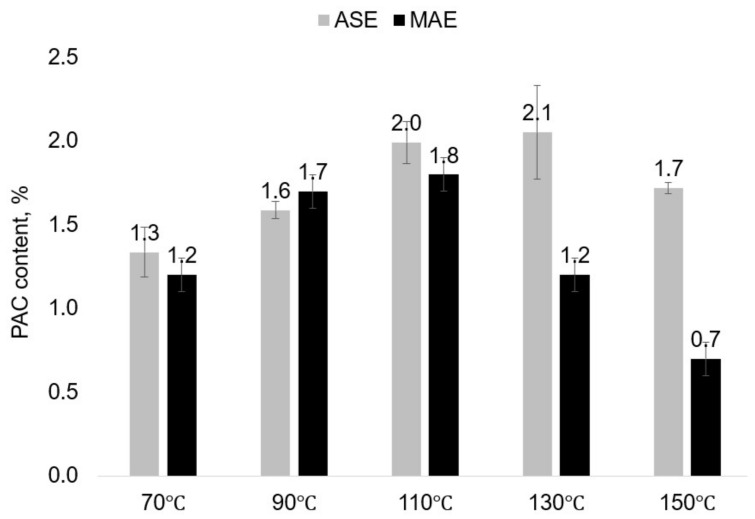
Proanthocyanidins (PAC) content in aspen bark water extracts obtained after 20 min of extraction depending on the extraction method and temperature (the error bars present the standard deviation of three repeated tests).

The obtained aspen bark water extracts contain a maximum of 2% of proantocyanidins corresponding to the 0.5% of proantocyanidins on the bark DM. The reported content of condensed tannins in the hybrid aspen bark was 0.30–0.57% of DM. [32]. At extraction temperatures up to 110 °C, both the ASE and MAE extracts have close contents of proantocyanidins; however, at higher extraction temperatures (130–150 °C), it is significantly lower in the MAE extracts (Figure 6).

### 2.4. Composition of Pine Bark Water Extracts

**Figure 7:**
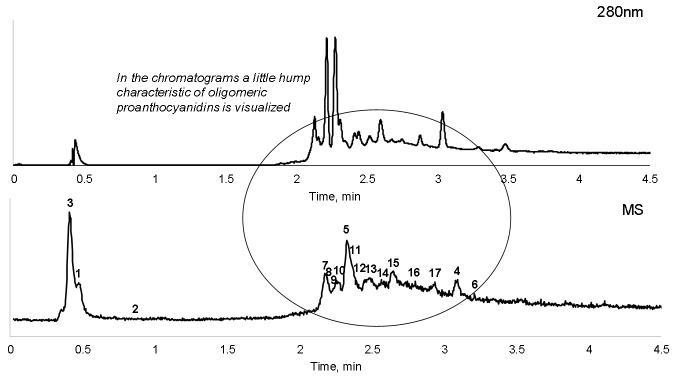
UV chromatogram at 280 nm and total ion chromatogram of the pine bark water extract obtained in microwave-assisted extraction at 90 °C, 5 min of isothermal heating (identification of the compounds is presented in Table 6).

In reversed-phase liquid chromatography applied for the analysis of pine bark water extracts, it is difficult to separate proanthocyanidins with a molecular weight exceeding that of the tetramers, and the respective elution order of the peak clusters is not necessarily correlated with the respective degree of polymerization [18]. Proanthocyanidin samples with a high polymerization degree generate a large unresolved hump in the chromatogram [44]. The hump typically is produced by large proanthocyanidin oligomers, mainly from decamers and higher polymers [45]. The chromatograms of the water extracts obtained by MAE visualized only a little hump, indicating the presence of oligomeric proanthocyanidins, mainly from dimers and decamers (Figure 7). Except for oligomeric proanthocyanidins, pine extract was shown to consist of quinic acid, protocatechuic acid, caffeic acid and the following flavonoids: catechin, taxifolin-O-hexoside and taxifolin (Table 6). These compounds are well-known constituents of pine bark extracts [11,18,21].

### 2.5. Effectivity of MAE in Terms of the Isolation of Proanthocyanidins, Flavonoids and Quinic Acid from Pine Bark

**Figure 8:**
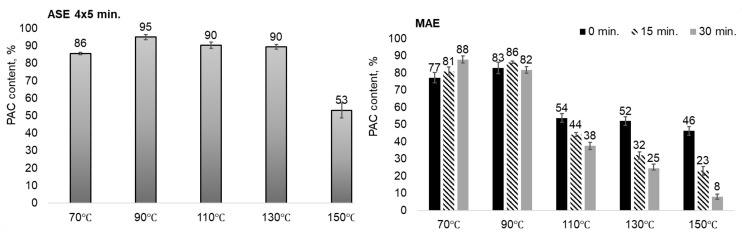
The content of proanthocyanidins in the pine bark water extracts depending on the extraction technique and conditions (the error bars present the standard deviation of three repeated tests).

In contrast to the aspen bark water extracts, oligomeric proanthocyanidins (PAC) are the dominating components of the pine bark water extracts, constituting up to 95% of the extracts (Figure 8). According to the literature, the maritime (*Pinus pinaster*) and *Pinus pinea* pine bark extracts obtained by methanol/water/acetic acid (49.5:49.5:1, *v*/*v*/*v*) extraction after the removal of lipophilic compounds contain 81.4 and 85.7% of PAC, but the commercial pine bark extract Pycnogenol^®^ contains 70.0% of PAC [21].

At 70–90 °C, the content of proanthocyanidins in the MAE extracts is close to that in the ASE extracts obtained at optimal extraction times (four extraction cycles of 5 min each). The MAE extracts obtained at 110–150 °C have a significantly lower PAC content, decreasing with the increasing time of isothermal heating.

Unfortunately, only a few proanthocyanidins were well separated by liquid chromatography and could be quantified on the basis of their UV peak area. Most proanthocyanidins had peaks that overlapped with other proanthocyanidins. Therefore, the selective ion monitoring (SIM) mode was used to select the molecular ions of the procyanidin groups (dimer, trimer and tetramer) in the pine bark extracts for their quantification. The oligomers from dimer to tetramer were quantified with the dimer calibration curve. The results were expressed as the percent of B2 dimer g equivalents content in the dry extracts. In contrast to the total proanthocyanidins content being significantly higher in the case of aspen bark ASE water extracts obtained at 130 °C and 90 °C vs. MAE extracts obtained at the same temperatures, the extracts obtained by MAE have a significantly higher content of proanthocyanidin dimers-tetramers (Table 7).

These data show that microwave-assisted extraction with dynamic heating, avoiding the isothermal heating step, promotes the isolation of proanthocyanidin dimers-tetramers from the pine bark by water.

**Figure 9:**
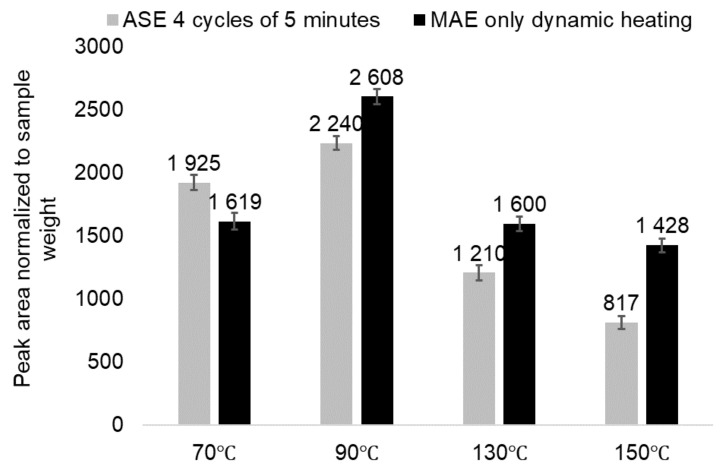
The comparative content of catechin in the pine bark water extracts depending on the extraction technique and temperature (the error bars present the standard deviation of three repeated tests).

At the same time, the extracts obtained by MAE at 90–130 °C after 0 min of isothermal heating contain more catechin in comparison to the ASE extracts with a higher content of total proanthocyanidins (Figure 9). This can be connected both with the promotion of catechin isolation from the bark as well as with the catechin formation being one of the products of tannins depolymerisation [16].

The MAE extraction does not promote the isolation of another flavonoid-taxifolin quantified by the application of commercial standards (Table 8).

Quinic acid and one B-type proanthocyanidin dimer, as important biologically active low-molecular components of pine bark water extracts, were also quantified using commercial standards (Table 8). Quinic acid was shown to have a positive influence on the antioxidant activity of the pine bark polar extracts. The reported content of quinic acid in the *Pinus pinaster* pine bark extract obtained by multi-step extraction with the application of dichloromethane for the removal of lipophilic compounds was 2.8% [21]. The application of microwave energy allowed for the obtention of the same content of quinic acid in the extract by the one-step water extraction of *Pinus sylvestris* bark (Table 8). The microwave-assisted extraction also promotes the isolation of the B2 proanthocyanidin dimer from pine bark, as, at the optimal extraction time, the MAE extracts obtained at different extraction temperatures contain more of this biologically active compound in comparison to the corresponding ASE extracts (Table 8).

### 2.6. The Biological Activity of the Aspen Bark and Pine Bark Water Extracts Obtained by Microwave-Assisted Extraction

In vitro screening studies for pharmacological activity may lead to the identification of new medicines, foods or dietary recommendations for the treatment or prevention of various ailments [46]. Xanthine oxidase, a versatile metalloflavoprotein enzyme that is found in the heart, lung and liver tissues, plays an important role in the metabolism of nucleic acids in vivo. Xanthine oxidase catalyzes the oxidative hydroxylation of hypoxanthine and xanthine to uric acid while generating reactive oxygen species. Therefore, this enzyme is a validated target for the therapeutic treatment of hyperuricemia caused by uric acid overproduction. The activity of xanthine oxidase and hyperuricemia have also been associated with a variety of conditions such as diabetes, hypertension and other cardiovascular diseases. Synthetic xanthine oxidase inhibitors, such as allopurinol, a purine derivative, and febuxostat, a thiazolecarboxylic acid derivative, exhibit significant therapeutic effects already present in the market. As an alternative to synthetic products with negative side effects such as renal toxicity and hypersensitivity reactions, several plant-derived compounds were considered as alternative xanthine-oxidase inhibitors [29,47,48]. The Proanthocyanidin-rich French maritime pine bark extract selectively inhibits xanthine oxidase [49]. The pine (*Pinus sylvestrys*) and aspen (*Populus tremula)* bark water extracts obtained by MAE during this work were tested as inhibitors of the xanthine oxidase enzyme. The extracts obtained at 70 °C after 30 min of isothermal heating were chosen, as they have the highest content of PAC in the case of pine bark extracts and of free salicin in the case of aspen bark extracts.

The xahntine oxidase inhibitory activity of the extracts was characterized as IC_50_ and IC_100_—the concentrations of the extracts (mg·L^−1^) needed to reduce the initial enzyme activity (50 mU·L^−1^) by 50% and 100%, respectively. The lower IC_50_ and IC_100_ values show a higher activity of extracts. The synthetic xanthine oxidase inhibitor, allopurinol, was used as a reference. Its IC_50_ in the test conditions was 0.11 mg·L^−1^.

**Figure 10:**
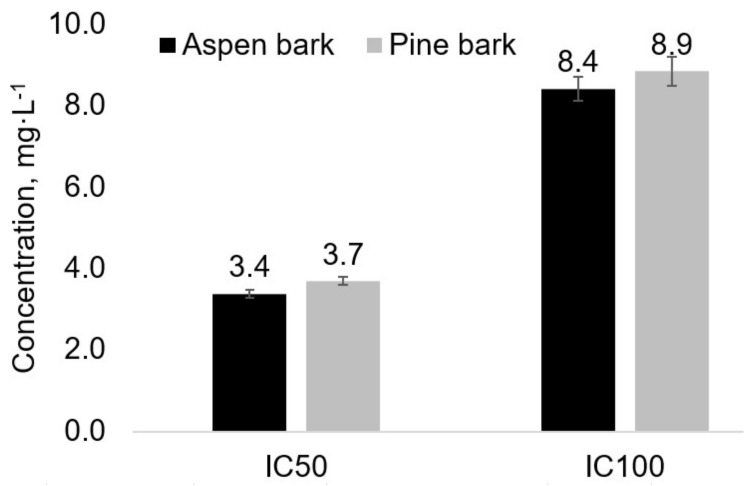
The xanthine oxidase inhibition activity of the aspen and pine bark water extracts obtained by microwave-assisted extraction (the error bars present the standard deviation of three repeated tests).

The IC_50_ of the aspen bark and pine bark extracts was 3.4 and 3.7 mg·L^−1^, respectively. The IC_100_ values of the aspen and pine bark extracts are also close (Figure 10). Taking into account that these are crude extracts consisting of a mixture of compounds, including a significant amount of carbohydrates and other inactive components, in comparison to pure synthetic allopurinol, the xanthine oxidase inhibition activity of the aspen and pine bark phenolic extractives can be evaluated as significant.

## 3. Materials and Methods

### 3.1. Plant Material

The raw material aspen (*Populus tremula*) bark was harvested in May 2019 from 30-year-old trees grown in the Talsu municipality in Latvia. They were grown in the Aegopodiosa forest in moderately moist soil. Another raw material pine (*Pinus silvestris* L.) bark was harvested in June 2019 from 23-year-old trees grown in the Smiltene municipality in Latvia. They were grown in moderately moist soil.

After collection, the bark was dried at room temperature until the moisture content was approximately 9%, and then it was milled using a Retch AS100 mill (Retch GmbH, Haan, Germany) at 1500 rpms with a 2 mm sieve. The milled bark was stored in a freezer at −18 °C until further analysis.

### 3.2. Chemicals and Reagents

The D-(-)-salicin (≥98.5% HPLC), D-(-)-quinic acid (≥98%), Procyanidin B2 (≥90% HPLC) and Taxifolin (≥85% HPLC) analytical standards were purchased from Sigma-Aldrich (Steinheim, Germany). The LC-MS hypergrade acetonitrile was purchased from Merck (LiChrosolv, Germany), and the formic acid (HiperSOLV Chromanorm) was purchased from VWR Chemicals. Milli-Q Type 1 ultrapure water (suitable for chromatography and other advanced analytical techniques) was used for sample preparation as well as the mobile phase. The Folin–Ciocalteu reagent, gallic acid, methanol and other chemicals used for the analyses were of analytical grade and were purchased from Sigma-Aldrich (Steinheim, Germany).

### 3.3. Composition Characterization of Bark Biomass

#### 3.3.1. Wet Chemistry Analysis

The ash content was measured as a residue after ignition at 550 ± 1 °C in a Carbolite ELF 11/6B furnace.

The content of acid insoluble lignin (Klason lignin) was determined by the Tappi Standard method, T 222 om -98. Correction on the dry ash free basis was done.

#### 3.3.2. Element Analysis

The elemental analysis (C, H, N) was conducted using a Vario MACRO elemental analyzer (ELEMENTAR Analysensysteme). The bark sample weight for the five repeated experiments varied in the range of 50–75 mg.

#### 3.3.3. Total Carbohydrates Content

The carbohydrates content, expressed as the monomeric sugar content after carbohydrates hydrolysis, was determined by the method of gas chromatography with flame ionization detection (GC-FID), as described elsewhere, with slight modifications [50]. About 10 mg of the sample was loaded into a screw tube, 0.125 mL of 72% H_2_SO_4_ was added and the mixture was incubated at room temperature for 3 h. Then, 3.5 mL of deionized water was added, and the mixture was incubated at 101 °C for 3 h. The cooled sample was neutralized with 0.32 mL of NH_4_OH, and 0.1 mL of the methyl-α-D-glucopyraniside solution (20 mg·mL^−1^) was added as an internal standard. In total, 0.2 mL of the sample was taken and 1 mL of 2% NaBH_4_ solution in dimethyl sulfoxide was added. The mixture was incubated at 40 °C for 90 min. The excess of NaBH_4_ was decomposed by adding 0.1 mL of glacial acetic acid. After that, 2 mL of acetic anhydride and 0.2 mL of 1-methylimidazole were added to the solution and incubated at 40 °C for 10 min. Then, 5 mL of water was added, and the solution was extracted with 1 mL of CH_2_Cl_2_ for 1 min. The CH_2_Cl_2_ layer was collected for GC analysis. The analysis was conducted using an Agilent 6850 series GC instrument with a 30 m DB-1701 column with an internal diameter of 0.25 mm and a film thickness of 0.25 µm. All of the results were expressed on the dry basis of the extract. All of the chemicals used for the analyses were purchased from Sigma-Aldrich, Steinheim, Germany.

#### 3.3.4. FTIR Spectroscopy

Fourier transform infrared (FTIR) spectra were recorded in KBr pellets by the Spectrum One apparatus (Perkin Elmer) by scanning from 500 to 4000 cm^−1^. The scan resolution and the number of scans were 4 cm^−1^ resolution and 64, respectively.

### 3.4. Microwave-Assisted Water Extraction

The microwave-assisted water extraction of the aspen and pine bark was performed using a microwave extraction device of original construction consisting of a circular coaxial wave guide with three magnetrons (output microwave power −0.850 kW per each magnetron), an extraction chamber (V = 1350 cm^3^), power supply units, a vacuum pump, a cooling section and a pressure relief valve [28,29]. A total of 108.50 g of bark with a water content of 9% and 550.00 g of deionized water (T = 18–20 °C) were loaded into the extraction chamber. The vacuum treatment of the suspension at the residual pressure of 50 mbar was done for 5 min to ensure the penetration of the solvent inside the biomass. Then, the atmospheric pressure in the chamber was recovered, and continual microwave heating with the rate of 25–35 °C·min^−1^ of the composition up to the desirable temperature in the range of 70–150 °C was performed, followed by automatically controlled isothermal heating for 5–30 min, which was achieved by the on/off action regime of the microwave generators. After the exposition of the biomass at a given temperature, the pressure inside the closed extraction chamber was removed by turning on the relief valve connected to the extraction chamber and the cooling section. After the 5 min exposition, the chamber was removed from the extraction column to be mounted into a screw-gearing press to separate the solid and liquid fractions. For each chosen temperature magnitude, one experiment was performed without the isothermal heating stage, e.g., a drop in pressure was achieved immediately after the achievement of the desirable temperature inside the extraction chamber. The liquid fraction enriched with extractives was filtered using a Buchner funnel, followed by lyophilic drying. The yields of the extractives were measured by weighing and were expressed as the percent on the dry basis of the non-treated bark.

The powder-like extracts were placed in a plastic box for freezing storage at −18 °C.

As the reference, the water extraction of the bark was performed using accelerated solvent extraction equipment (ASE 350 apparatus, Thermo scientific, Dionex, Sunnyvale, CA, USA) [51,52]. In total, 40.00 g of the pine or aspen barks was loaded into the extraction cell. The extraction was performed at a 105 atm pressure in the atmosphere of nitrogen at different temperatures (70–150 °C), the extraction static cycle time was 5 min and the number of extraction static cycles was four.

### 3.5. Analytical Pyrolysis

The Py-GC/MS/FID analysis of the bark and bark extracts was performed using a Micro Double-shot Pyrolyzer Py-3030D (Frontier Laboratories. Ltd., Fukushima, Japan) (pyrolysis temperature, 500 °C; heating rate, 600 °C·s^−1^) directly coupled with a Shimadzu GC/MS/FID-QP ULTRA 2010 apparatus (Kyoto, Japan) equipped with a capillary column RTX-1701 (Restec, Metairie, LA, USA) and a 60 m × 0.25 mm × 0.25 mm film (an injector temperature of 250 °C; an ion source with an EI of 70 eV; an MS scan range *m*/*z* of 15–350; carrier gas helium at a flow rate of 1 mL·min^−1^; and a split ratio of 1:30). The mass of the sample probe (residual moisture content <1%) was 1.00–2.00 mg. The oven program was as follows: 1 min isothermal at 60 °C, followed by 6 °C·min^−1^ to 270 °C and the final hold at 270 °C for 10 min. The mass spectrometer was operated in electron impact mode using 70 eV electron energy. The identification of the individual compounds was performed based on GC/MS chromatography using Library MS NIST 11 and NIST 11 s, whereas the relative area of the peak of the individual compounds (% from chromatogram) was calculated using Shimadzu software based on the GC/FID data.

### 3.6. Analysis of Extracts by Liquid Chromatography-Mass Spectrometry

Lyophilized water extracts were dissolved in 50% acetonitrile (2 mg·mL^−1^). They were filtered (Nylon filter, 0.45 μm pore size) and used for experiments. The analyses were performed on an Acquity UHPLC system with a UV detector (Waters Corp.) that was coupled with a quadrupole-time of flight MS instrument (Synapt Q-TOF MS, Waters) with an electrospray ionization source. The separation was carried out on a UHPLC column (2.1 mm × 50 mm i.d., 1.7 µm, BEH C18) (Waters Acquity) at a flow rate of 0.35 mL·min^−1^. The eluents were 0.1% formic acid in water (A) and acetonitrile (B). A gradient solvent system was used: 0–7 min, 5–95% (B); 7–8 min, 95–95% (B); 8–9 min, 95–5% (B). The injection volume was 2 μL. The major operating parameters for the Q-TOF MS were: capillary voltage, 2.2 kV (−); cone voltage, 60 V; cone gas flow, 100 L∙h^−1^; collision energy, 6 eV; source temperature, 120 °C; desolvation temperature, 450 °C; collision gas, argon; desolvation gas, nitrogen; flow rate, 800 L∙h^−1^; data acquisition range, *m*/*z* 50–1200 Da; ionization mode, negative. The compounds were tentatively identified and compared to those reported in the literature and confirmed through databases—specifically the GNPS and ChemSpider, focusing on MS/MS fragmentation patterns and accurate mass.

#### 3.6.1. Total Salicin Derivatives and Free Salicin Content

The quantitative liquid chromatography analysis of salicin (and its derivatives) was basically performed in accordance with the method described in the European Pharmacopoeia [53], with certain modifications according to the following protocols.

#### 3.6.2. Alkaline Hydrolysis of Extracts

The lyophilized extracts of the aspen bark (~50 mg) were taken up with 5.0 mL of methanol; 2.0 mL of 0.1 M sodium hydroxide were added and then heated in a water bath at 60 °C with frequent shaking for 1.5 h [42]. After cooling, the hydrolysate was neutralized with 1 M H_2_SO_4_ and then made up to volume of 10 mL in the volumetric flask with methanol. Then, 1 mL of this solution was filtered (Nylon filter, 0.45 μm pore size) and then used for the UHPLC-ESI-MS/MS analysis of salicin. The alkaline hydrolysis produced a solution containing free salicin and its derivatives, which were hydrolyzed to salicin. “Total salicin” means salicin derivatives expressed as salicin.

#### 3.6.3. Free Salicin Analysis

The extracts were dissolved in methanol with an approximate concentration of 5–8 mg·mL^−1^ and filtered (Nylon filter, 0.45 μm pore size); then, they were used for the UHPLC-ESI-MS/MS experiments. The peak areas were recorded, and the amount of salicin was calculated using the calibration plot on UHPLC. Approximately 10 mg of salicin was dissolved into 10 mL of methanol. The dilutions prepared by stock solution and for UHPLC linearity were determined by seven different concentrations of salicin in triplicate, and the calibration curve was plotted in a range from 0.04–0.91 mg·mL^−1^ of salicin. The calibration curve was plotted by replicate analysis at all of the concentration levels, and the linear relationship was evaluated using the least square method with the Microsoft^®^ Excel program. The regression equation and the coefficient of determination (R^2^) of salicin for UHPLC were Y = 9750.5 x + 62.249, 0.9993, respectively. The absorption maxima of salicin was detected at 267 nm.

#### 3.6.4. Quantification of Taxifolin and Procyanidin B2

The quantitative analyses were carried out with the previously described UHPLC-ESI-MS/MS equipment, using taxifolin and procyanidin B2 as the external standards. The separation was carried out with a UHPLC column (2.1 × 50 mm i.d., 1.7 µm, BEHC18) (Waters Acquity) at a constant temperature of 30 °C. The mobile phase was composed of an aqueous solution of 0.1% formic acid (A) and acetonitrile (B), and the program of the mobile phase was as follows: 0 min, 5% B; 5 min, 20% B; 6 min, 75% B; 7 min, 20% B; 8 min, 5% B at a flow rate of 0.35 mL·min^−1^ with a 2 μL sample injection volume. The analytes were monitored using negative selected ion monitoring (SIM) *m*/*z* 285 for Taxifolin [M-H]^−^ and *m*/*z* 577 for Procyanidin B2 [M-H]^−^. The stock solutions of Taxifolin and Procyanidin B2 were prepared by dissolving the reference standards in 50% (*v*/*v*) acetonitrile at final concentrations of 0.26, and 0.10 mg·mL^−1^, respectively. The standard working solutions were prepared by diluting with 50% (*v*/*v*) acetonitrile to the desired concentrations. The chromatograms were recorded at 280 nm. The calibration curves were constructed by plotting the peak areas against the corresponding mass concentrations.

#### 3.6.5. Quantification of Proanthocyanidin Dimers to Tetramers

The analyses were carried out with the previously described UHPLC-ESI-MS/MS equipment. The pine bark extracts were dissolved in the 50% (*v*/*v*) acetonitrile and filtered. The selective ion monitoring (SIM) mode was used to select the molecular ions of the procyanidin groups (dimer, trimer and tetramer) in the pine bark extracts for their quantification. The analytes were monitored using negative selected ion monitoring (SIM) *m*/*z* 577 for the procyanidin dimers [M-H]^−^, *m*/*z* 865 for the procyanidin trimers [M-H]^−^ and *m*/*z* 1153 for the procyanidin tetramers. The oligomers from dimer to tetramer were quantified with the procyanidin B2 calibration curve. The stock solution and each dilution were injected into the column three times and used to construct a calibration curve. The results were expressed as the percentages of B2 dimer equivalents of the extract.

#### 3.6.6. Detection of Catechin Content

The catechin was detected with the previously described UHPLC-ESI-MS/MS equipment. The pine bark extracts were dissolved in the 50% (*v*/*v*) acetonitrile and filtered. The selective ion monitoring (SIM) mode *m*/*z* 289 was used to select the molecular ion of the catechin in the pine bark extracts. The catechin peak area was normalized to the sample weight.

#### 3.6.7. Quantification of Quinic Acid

The quantitative analysis was carried out with the previously described UHPLC-ESI-MS/MS equipment, using quinic acid as the external standard. The calibration standard solutions were prepared using quinic acid (≥98%, Sigma Aldrich, Steinheim, Germany) at concentrations of 230, 170, 115, 58, 29 and 15 µg·mL^−1^ as stock solutions in 50% gradient grade acetonitrile. Each standard solution was prepared in duplicate and injected once. The solutions were analyzed in the MRM mode, and the observed peak area ratios were plotted vs. the concentration ratios. For the quantification of quinic acid, the MRM transition from the deprotonated molecular ion [M-H]^–^ *m*/*z* 191 to *m*/*z* 85 was used as the quantifier. The peak areas were recorded, and the amount of quinic acid was calculated using the calibration.

### 3.7. Total Phenolics Content

The content of the polyphenolics in the extracts was determined by Folin–Ciocalteu analysis according to the procedure described by [54]. An total of 1 mL of hydrophilic extractives solution (in 50% (*v*:*v*) ethanol) was added to 0.5 mL of the Folin–Ciocalteu phenol reagent followed by gentle shaking. After 5 min, 1 mL of 20% (*w*/*v*) sodium carbonate was added. The solution was immediately diluted up to 5 mL with distilled water and mixed thoroughly. After 10 min, an optical density at 765 nm of the resulting blue complex was measured using a PerkinElmer Lambda 650 UV/VIS spectrophotometer against the blank, using gallic acid as the standard. The total phenolic contents were expressed as the g of gallic acid equivalents (GAE) per g of the dry extract sample.

### 3.8. Total Flavonoids Content

The total flavonoid content was calculated using zirconium chloride (ZrOCl_2_·8H_2_O) according to the procedure described by [55]. Aliquots (1 mL) of the aqua extracts were placed in two test tubes, respectively. A total of 7 mL of methanol was added to one tube. In the other tube, 1 mL of 2% ZrOCl_2_·8H_2_O and 6 mL of methanol was added. The solution was mixed again and placed into a water bath at 30 °C for 1 h. The absorbance was measured at 420 nm with the PerkinElmer Lambda 650 UV/VIS spectrophotometer, and Δ was calculated. The amount of total flavonoids was calculated as a rutin equivalent from the standard curve and expressed as the mg rutin per g of dry extract (mg·g^−1^).

### 3.9. Total Proanthocyanidins Content

The acid-butanol method for determining the total proanthocyanidin content in the extracts was performed, as described in [55]. In a screw cap tube, 1 mL of the sample, 6 mL of the acid-butanol reagent (950 mL n-butanol with 50 mL conc. HCl) and 0.2 mL of the 2% ferric ammonium sulfate solution were added in 2 mol HCl. The samples were vortexed and incubated for 50 min in a 90 °C degree water bath. Afterwards, the samples were cooled, and the absorbance was measured at 550 nm. The concentrations of proanthocyanidins were determined by the calibration curve of the B2 epicatechin dimer standard.

### 3.10. Xanthine Oxidase Inhibition Activity

The activity of xanthine oxidase was measured using a commercial fluorometric assay kit (Cayman Chemical Co., Ann Arbor, MI, USA, item number 10010895) following the manufacturer’s instructions, with the following difference: instead of 50 µL of the blood serum supernatant being collected with the help of the buffer solution, 50 µL of the prepared xanthine-oxidase and inhibitor mixture was used to react with 50 µL of the “assay cocktail”, prepared according to the provided protocol. The enzyme-inhibitor mixture was prepared by the addition of 10, 20, 50 or 100 µL of the bark water extract solution (50 mg·L^−1^ in DMSO) or allopurinol solution (1 mg·L^−1^ in DMSO) to 20 µL of the xanthine oxidase stock solution containing 1 mU·mL^−1^ of enzyme, prepared as described in the kit protocol, and the addition of the provided “sample buffer” (diluted as described in the protocol) to the total volume of 200 µL (170, 160, 130 and 80 µL of the “sample buffer” was added depending on the inhibitor solution volume). The fluorescence of the reaction mixture was measured using a multiwell plate reader (Synergy HT) with 530/25 nm excitation and 590/35 nm emission. The inhibitor solutions in the “sample buffer” of the concentrations corresponding to their concentration in the prepared enzyme-inhibitor mixture were used instead of the blank sample buffer to correct the fluorescence, as described in the kit protocol. The xanthine oxidase activity was calculated using the xanthine standard curve according to the protocol.

## 4. Conclusions

Aspen (*Populus tremula*) and pine (*Pinus sylvestris*) bark biomass of about one fourth and one seventh, respectively, are composed of extractives, the main part of which can be isolated by one-step water MAE. The dominating constituents of aspen bark water extracts are biologically active phenyl glycosides, including salicin and its derivatives. The one-cycle microwave-assisted extraction, specially at 70 °C after 30 min of isothermal heating, promoted the isolation of salicin from the aspen bark, allowing for the obtention of a two-times-higher free salicin concentration in the water extracts (−14% vs. 7%) reached by the four-cycle accelerated solvent extraction. The major constituents of the pine bark water extracts are oligomeric proanthocyanidins, mainly from dimers to decamers, constituting up to 88% of the extracts obtained by microwave-assisted extraction and 95% of those obtained by the accelerated solvent extraction. The microwave-assisted extraction of pine bark with high rate (25–35 °C·min^−1^) dynamic heating to 90–130 °C, avoiding the isothermal step and using the single solvent portion, allowed for the obtention of a 1.7-times-higher concentration of proanthocyanidin dimers-tetramers, a 1.3-times-higher concentration of catechin and a 1.2-times-higher concentration of quinic acid in the water extracts in comparison to a longer and more solvent-consuming accelerated solvent extraction performed at the same temperature. The xanthine oxidase inhibition activity of the aspen and pine bark phenolic extractives was evaluated as significant, with IC_50_ values of the crude extracts containing inactive admixtures equal to 3.4–3.7 mg·L^−1^ vs. 0.11 mg·L^−1^ of the pure synthetic xanthine oxidase inhibitor allopurinol. The results of this work open new opportunities for the production of plant-based therapeutic agents, as well as for the development of efficient conversion strategies of waste lignocellulosic biomass.

## Figures and Tables

**Table 1:** The chemical composition of aspen (*Populus tremula*) and pine (*Pinus sylvestris* L.) bark.

Feedstock	Content, % on DM			
	Ash ^1^	Carbohydrates Total ^1^	Klason Lignin Acid Insoluble ^2^	Extractives ^3^
Aspen bark	3.77 ± 0.10	43.6 ± 1.2	26.1 ± 1.1	26.5 ± 0.7
Pine bark	1.79 ± 0.05	42.3 ± 0.8	41.8 ± 1.8	14.1 ± 0.3

^1^ Experimental data, the relative standard deviation of three repeated tests was ≤3%; ^2^ experimental data, the relative standard deviation of three repeated tests was ≤5%; ^3^ calculated by equation. Extractives (%) = 100–ash–carbohydrates total–Klason lignin acid insoluble.

**Table 2:** Element composition of aspen (*Populus tremula*) and pine (*Pinus sylvestris* L.) bark on DM vs. that of oil products.

Feedstock	Content, % on Ash Free DM ^1^			
	C	H	O ^2^	N
Aspen bark	51.53 ± 1.34	5.40 ± 0.16	42.36 ± 0.81	0.71 ± 0.01
Pine bark	52.94 ± 1.32	5.07 ± 0.11	41.53 ± 1.21	0.46 ± 0.01
Oil products [6]	83–87	10–14	0.1–1.5	0.1–2

^1^ The relative standard deviation for three repeated tests was ≤3%; ^2^ calculated by difference.

**Table 3:** The yield (%) of bark extractives depending on trees species, extraction solvent and procedure.

Bark Source	Solvent, Extraction Conditions	Procedure	Extractives Yield, % on Bark D.M
Hybrid aspen (*Populus tremula* L. × *tremuloides Michx*.)	Acetone (95%, aq.)	Accelerated solvent extraction, 100 °C, 3 cycles of 15 min each	16.2–23.9% [32]
Quaking aspen (*Populus tremuloides*)	Acetone (99.8%)	Soxhlet system for 7 h over 6 cycles of extraction	≈14.5% [37]
Quaking aspen (*Populus tremuloides*)	Distilled water	Accelerated solvent extraction, 100 °C, 6 cycles of 10 min each, 1500 psi	≈15% [37]
Quaking aspen (*Populus tremuloides*)	Methanol (99.9%)	Accelerated solvent extraction, 100 °C, 6 cycles of 10 min each, 1500 psi	≈19% [37]
Quaking aspen (*Populus tremuloides*)	Denaturised ethanol	Soxhlet system for 7 h over 6 cycles of extraction	≈18% [37]
*Pinus sylvestris*	Acetone:water (95:5; *v*/*v*) → water (100%)	Sequential accelerated solvent extraction, 150 °C, 3 static cycles of 5 min each, 2000 psi	19.4% (acetone 5.5% + water 13.9%) [38]
*Pinus sylvestris*	Hot water	TAPPI 1993a	18.72% [39]
*Pinus sylvestris*	Alcohol	TAPPI 1988a	18.33% [39]
*Pinus sylvestris* L.	Methanol (80%)	Extraction using a magnetic stirrer, 3 cycles of 3 h each	17.35% [11]

**Table 4:** Tentative identification of compounds in the aspen bark MAE water extract.

Peak	[M-H]^−^	Fragments	Compounds	Compound Class	Ret. Time, min
1	331	285, 123	Salicin	Phenolic Glycosides	1.62
2	373	327	2O-acetyl-salicin		2.37
3	423	469; 317	Salicortin		2.86
4	405	451; 283	Salicyloyl-salicin		3.62
5	527	573	Tremulacin		6.67
6	435	389; 121	Tremuloidin		5.05
7	477	285; 119	Kaempferol-hexoside		2.98
9	447	285	Luteolin-hexoside		3.74
10	487	307	Acetylglycitin		3.14
11	423	307; 259; 145; 163	Grandidentatin		3.19
12	435	273; 163	Phloridzin		4.07
13	423	307; 259; 163; 145	Grandidentatin		4.62
14	431	269	Apigenin-7-O-glucoside		5.97
15	191	173, 111, 67	Citric acid derivative	Phenolic acids	0.47
16	325	163	Coumaric acid glucoside		2.05
17	315	300/299	Isorhamnetin	Flavonoids	1.33
18	303	285, 285, 247, 111	Ouercetin		2.96
19	521	423; 373	Trilobolide	Terpenoid	2.49
20	683	341; 281; 179, 161, 135	Caffeic acid hexose and Hexose polymer	Phenolic acids/sugars	0.40

**Table 5:** Free and total salicin content (%) in water extracts isolated from aspen bark by MAE depending on the extraction conditions.

Extraction Conditions ^1^	Content of Salicin, (%) on DM of Extract ^2^		Content of Salicin, (%) on DM of Bark ^3^	
	Free	Total	Free	Total
70 °C; 5 min.	9.42 ± 0.21	16.52 ± 0.36	1.74 ± 0.05	2.92 ± 0.09
70 °C; 20 min.	8.23 ± 0.18	12.07 ± 0.31	1.41 ± 0.05	2.11 ± 0.06
70 °C; 30 min.	9.74 ± 0.17	19.33 ± 0.38	1.76 ± 0.06	3.53 ± 0.15
90 °C; 5 min.	8.51 ± 0.19	18.71 ± 0.47	1.55 ± 0.03	3.92 ± 0.17
90 °C; 20 min.	9.63 ± 0.22	18.12 ± 0.27	1.84 ± 0.05	3.37 ± 0.14
90 °C; 30 min.	10.48 ± 0.28	19.21 ± 0.23	2.41 ± 0.06	3.63 ± 0.13
110 °C, 5 min.	10.71 ± 0.17	19.04 ± 0.39	2.08 ± 0.05	3.68 ± 0.14
110 °C; 20 min.	14.05 ± 0.33	17.64 ± 0.42	2.73 ± 0.11	3.42 ± 0.17
110 °C; 30 min.	11.07 ± 0.21	16.13 ± 0.34	2.23 ± 0.08	3.24 ± 0.12
130 °C; 5 min.	10.81 ± 0.25	17.32 ± 0.39	2.25 ± 0.05	3.57 ± 0.14
130 °C; 30 min.	12.15 ± 0.27	15.38 ± 0.29	2.99 ± 0.08	3.72 ± 0.16
150 °C; 5 min.	9.94 ± 0.14	13.71 ± 0.22	2.51 ± 0.09	3.52 ± 0.12
150 °C; 30 min.	10.32 ± 0.29	13.62 ± 0.34	2.82 ± 0.07	3.77 ± 0.16

^1^ Isothermal heating; ^2^ the relative standard deviation for three repeated tests was ≤3.0%; ^3^ the relative standard deviation for three repeated tests was ≤5.0%.

**Table 6:** Pine bark extract tentative identification.

Peak	[M-H]^−^	Fragments	Compounds	Compound Class	Ret.time, min
1	191	173, 127, 111, 85	Quinic acid	Phenolic acids	0.47
2	299	137, 93	P-Hydroxy benzoic acid hexoside		0.86
3	341	179, 161, 119, 101,	Sucrose	Carbohydrates	0.41
4	465	447, 437, 259, 285, 125	Taxifolin-O-hexoside	Flavonoids	3.09
5	289	245	Catechin or epicatechin		2.33
6	303	285, 177, 125	Taxifolin		3.23
7	577	559, 461, 425, 289, 245, 203	B-type proanthocyanidin dimer	Proanthocyanidins	2.17
8	1153	865, 577, 425,	B-type proanthocyanidin tetramer		2.20
9	865	739, 577, 543, 425, 289, 245	B-type proanthocyanidin trimer		2.26
10	865	822, 793, 713, 577	B-type proanthocyanidin trimer isomer		2.28
11	1153	863, 575, 287	B-type proanthocyanidin tetramer isomer		2.39
12	1153	865, 575, 289, 245	B-type proanthocyanidin tetramer isomer		2.43
13	1153	1008, 865, 577, 289, 245	B-type proanthocyanidin tetramer isomer		2.48
14	865	577, 289, 245	B-type proanthocyanidin trimer isomer		2.50
15	1153	865, 577, 289, 245	B-type proanthocyanidin tetramer isomer		2.56
16	1153	865, 577, 289, 245	B-type proanthocyanidin tetramer isomer		2.58
18	1153	864, 491, 315, 289	B-type proanthocyanidin tetramer isomer		2.62

**Table 7:** Content of the proanthocyanidin dimers, trimers and teratmers calculated as the procyanidin B2 equivalents, %.

Extraction Conditions	Dimers, % *	Trimers, % *	Tetramers, % *	Total Dimers-Tetramers, % *
ASE 90 °C (4 × 5 min.)	0.98 ± 0.02	2.02 ± 0.05	0.54 ± 0.01	3.54 ± 0.07
MAE 90 °C 0 min of isothermal heating	1.19 ± 0.03	2.64 ± 0.07	0.57 ± 0.01	4.40 ± 0.09
ASE 130 °C (4 × 5 min.)	0.81 ± 0.02	1.12 ± 0.03	0.52 ± 0.02	2.44 ± 0.07
MAE 130 °C 0 min of isothermal heating	0.92 ± 0.02	2.93 ± 0.07	0.36 ± 0.01	4.21 ± 0.11

* The relative standard deviation for three repeated tests was ≤3.0%.

**Table 8:** Quinic acid, taxifolin and proanthocyanidin B2 dimer content in pine extracts.

Extraction Conditions	Quinic Acid, % *	Taxifolin, % *	B2 Dimer, % *
MAE 70 °C 0 min. of isothermal heating	2.56 ± 0.07	0.10 ± 0.003	0.80 ± 0.02
ASE 70 °C (4 × 5 min.)	2.09 ± 0.05	0.13 ± 0.003	0.80 ± 0.02
MAE 90 °C 0 min. of isothermal heating	2.80 ± 0.08	0.21 ± 0.005	1.19 ± 0.03
ASE 90 °C (4 × 5 min.)	2.29 ± 0.07	0.54 ± 0.015	0.98 ± 0.03
MAE 130 °C 0 min. of isothermal heating	0.17 ± 0.004	0.12 ± 0.002	0.92 ± 0.02
ASE 130 °C (4 × 5 min.)	0.13 ± 0.003	0.24 ± 0.005	0.81 ± 0.02
MAE 150 °C 0 min. of isothermal heating	0.80 ± 0.02	0.01 ± 0.0003	1.18 ± 0.03
ASE 150 °C (4 × 5 min.)	0.23 ± 0.005	0.02 ± 0.0003	0.44 ± 0.01

* The relative standard deviation for three repeated tests was ≤3.0%.

## Data Availability

Not applicable.
